# Supplementary material for: The chromatin remodeler Chd1 supports MRX and Exo1 functions in resection of DNA double-strand breaks
Source: PLoS Genet. 2021 Sep 14;17(9):e1009807. doi: 10.1371/journal.pgen.1009807 (PMC8462745; doi:10.1371/journal.pgen.1009807)
Supplement: S1 Table — (DOCX) [file pgen.1009807.s001.docx]

**S1 Table. *S. cerevisiae* strains used in this study.**

| Strain | Relevant genotype | Source |
| --- | --- | --- |
| W303 | *MAT*a/α *ade2-1 can1-100 his3-11,15 leu2-3,112 trp1-1 ura3-1 rad5-535* |  |
| DMP7292/2A | W303 *MAT*a *chd1∆::HPHMX* | This study |
| DMP7252/3A | W303 *MAT*a *chd1-E514A::LEU2* | This study |
| DMP7289/2D | W303 *MAT*a *chd1-K407R::LEU2* | This study |
| DMP5781/1B | W303 *MAT*a *rad50-V1269M::KANMX* | 1 |
| DMP7145/1D | W303 *MAT*a *chd1∆::HPHMX rad50-V1269M::KANMX* | This study |
| DMP7252/3B | W303 *MAT*a *chd1-E514A::LEU2 rad50-V1269M::KANMX* | This study |
| DMP7289/6B | W303 *MAT*a *chd1-K407R::LEU2 rad50-V1269M::KANMX* | This study |
| YLL2479.1 | W303 *MAT*a *dnl4∆::KANMX* | This study |
| JKM139 | *MAT*a *hml∆*::*ADE1,* *hmr∆::ADE1*, *ade1-100, lys5,*  *leu2-3,112, trp1::hisG ura3-52, ho, ade3::GAL-HO site* | 2 |
| YLL4201.5 | JKM139 *MAT*a *chd1∆::HPHMX* | This study |
| YLL4350.1 | JKM139 *MAT*a *chd1-E514A::LEU2* | This study |
| YLL4351.1 | JKM139 *MAT*a *chd1-K407R::LEU2* | This study |
| YLL3077.12 | JKM139 *MAT*a *rad51∆::HPHMX* | This study |
| YLL1769.1 | JKM139 *MAT*a *mre11∆::NATMX* | 3 |
| YLL1523.3 | JKM139 *MAT*a *sae2∆::KANMX* | 4 |
| YLL4291.4 | JKM139 *MAT*a *CHD1-18MYC::URA3* | This study |
| YLL1854.2 | JKM139 *MAT*a *MRE11-18MYC::TRP1* | 1 |
| DMP7076/1D | JKM139 *MAT*a *MRE11-18MYC::TRP1* *chd1∆::HPHMX* | This study |
| DMP7311/8D | JKM139 *MAT*a *MRE11-18MYC::TRP1* *chd1-E514A::LEU2* | This study |
| DMP7312/4D | JKM139 *MAT*a *MRE11-18MYC::TRP1* *chd1-K407R::LEU2* | This study |
| DMP6021/4A | JKM139 *MAT*a *MRE11-18MYC::TRP1* *rad50-V1269M::KANMX* | 1 |
| DMP7373/9B | JKM139 *MAT*a *MRE11-18MYC::TRP1* *rad50-V1269M::KANMX chd1∆::HPHMX* | This study |
| YLL1643.1 | JKM139 *MAT*a *bar1∆::HPHMX* | 5 |
| DMP6433/6C | JKM139 *MAT*a *bar1∆::TRP1 YKU70-3HA::URA3* | 5 |
| DMP7194/2B | JKM139 *MAT*a *bar1∆::TRP1 YKU70-3HA::URA3 chd1∆::HPHMX* | This study |
| DMP7251/10A | JKM139 *MAT*a *bar1∆::TRP1 YKU70-3HA::URA3 chd1-E514A::LEU2* | This study |
| DMP5819/3D | JKM139 *MAT*a *rad50-V1269M::KANMX* | 1 |
| DMP7094/3B | JKM139 *MAT*a *rad50-V1269M::KANMX chd1∆::HPHMX* | This study |
| YLL1959.2 | JKM139 *MAT*a *EXO1-18MYC::TRP1* | 3 |
| DMP7385/4A | JKM139 *MAT*a *EXO1-18MYC::TRP1 chd1∆::HPHMX* | This study |
| DMP7387/6D | JKM139 *MAT*a *EXO1-18MYC::TRP1 chd1-K407R::LEU2* | This study |
| DMP7386/10C | JKM139 *MAT*a *EXO1-18MYC::TRP1 chd1-E514A::LEU2* | This study |
| YLL3136.13 | JKM139 *MAT*a *SAE2-18MYC::TRP1* | This study |
| YLL4232.5 | JKM139 *MAT*a *SAE2-18MYC::TRP1 chd1∆::HPHMX* | This study |
| DMP7309/5D | JKM139 *MAT*a *SAE2-18MYC::TRP1 chd1-E514A::LEU2* | This study |
| DMP7310/1C | JKM139 *MAT*a *SAE2-18MYC::TRP1 chd1-K407R::LEU2* | This study |
| YMV45 | *ho hml::ADE1 mata::hisG hmr::ADE1 leu2::leu2(Asp718-SalI)-URA3-*pBR332*-MATa ade3::GAL::HO ade1 lys5 ura3-52 trp1::hisG* | 6 |
| YLL4304.3 | YMV45 *chd1∆::TRP1* | This study |
| YLL4329.1 | YMV45 *chd1-E514A::LEU2* | This study |
| YLL4394.1 | YMV45 *chd1-K407R::LEU2* | This study |
| YLL4339.6 | YMV45 *rad52∆::HPHMX* | This study |
| YLL4307.3 | YMV45 *rad52∆::HPHMX chd1∆::TRP1* | This study |
| YLL4338.1 | YMV45 *rad52∆::HPHMX chd1-E514A::LEU2* | This study |
| YLL4395.1 | YMV45 *rad52∆::HPHMX chd1-K407R::LEU2* | This study |
| YLL4405.1 | YMV45 *rad50-V1269M::KANMX* | This study |
| YLL4406.1 | YMV45 *rad50-V1269M::KANMX chd1∆::TRP1* | This study |
| tGI354 | *ho hml∆::ADE1 MATa-inc hmr∆::ADE1 ade1 leu2-3;112 lys5 trp1::hisG ura3-52 ade3::GAL::HO arg5,6::MATa::HPHMX* | 7 |
| YLL4306.6 | tGI354 *chd1∆::TRP1* | This study |
| YLL4330.1 | tGI354 *chd1-E514A::LEU2* | This study |
| YLL4403.38 | tGI354 *rad50-V1269M::KANMX* | This study |
| YLL4404.23 | tGI354 *rad50-V1269M::KANMX chd1∆::TRP1* | This study |

**References**

1. Cassani C, Gobbini E, Wang W, Niu H, Clerici M, Sung P, Longhese MP. Tel1 and Rif2 regulate MRX functions in end-tethering and repair of DNA double-strand breaks. PLoS Biol. 2016;14, e1002387.

2. Lee SE, Moore JK, Holmes A, Umezu K, Kolodner RD, Haber JE. *Saccharomyces* Ku70, Mre11/Rad50 and RPA proteins regulate adaptation to G2/M arrest after DNA damage. Cell. 1998;94, 399-409.

3. Manfrini N, Trovesi C, Wery M, Martina M, Cesena D, Descrimes M, Morillon A, d'Adda di Fagagna F, Longhese MP. RNA-processing proteins regulate Mec1/ATR activation by promoting generation of RPA-coated ssDNA. EMBO Rep. 2015;16, 221-31.

4. Gobbini E, Villa M, Gnugnoli M, Menin L, Clerici M, Longhese MP. Sae2 function at DNA double-strand breaks is bypassed by dampening Tel1 or Rad53 Activity. PLoS Genet. 2015;11, e1005685.

5. Gobbini E, Cassani C, Vertemara J, Wang W, Mambretti F, Casari E, Sung P, Tisi R, Zampella G, Longhese MP. The MRX complex regulates Exo1 resection activity by altering DNA end structure. EMBO J. 2018;37, e98588.

6. Vaze MB, Pellicioli A, Lee SE, Ira G, Liberi G, Arbel-Eden A, Foiani M, Haber JE. Recovery from checkpoint-mediated arrest after repair of a double-strand break requires Srs2 helicase. Mol Cell. 2002;10, 373-385.

7. Saponaro M, Callahan D, Zheng X, Krejci L, Haber JE, Klein HL, Liberi G. Cdk1 targets Srs2 to complete synthesis-dependent strand annealing and to promote recombinational repair. PLoS Genet. 2010;6, e1000858.
