# Supplementary material for: The chromatin remodeler Chd1 supports MRX and Exo1 functions in resection of DNA double-strand breaks
Source: PLoS Genet. 2021 Sep 14;17(9):e1009807. doi: 10.1371/journal.pgen.1009807 (PMC8462745; doi:10.1371/journal.pgen.1009807)
Supplement: S2 Table — (DOCX) [file pgen.1009807.s002.docx]

**S2 Table. Oligonucleotides used in this study.**

| **Name** | **Sequence (5’-3’)** | **Experiment** |
| --- | --- | --- |
| ARO- | ACCTACAGGAGGACCCGAAA | ChIP |
| ARO+ | TGAGTCGTTACAAGGTGATGCC | ChIP |
| MAT 0.2- | CCCGTATAGCCAATTCGTTC | ChIP |
| MAT 0.2+ | TCAGACTCAAGCAAACAATCAA | ChIP |
| MAT 0.6- | CATGCGGTTCACATGACTTT | ChIP |
| MAT 0.6+ | CACCCAAGAAGGCGAATAAG | ChIP |
| MAT 1.8- | CGCGAGTCTTATGCCAAAAA | ChIP |
| MAT 1.8+ | ACGTCGTTGTTAATGGTGGTG | ChIP |
| MAT 5.4- | GGACGACTTTAAGATGGAAGGA | ChIP |
| MAT 5.4+ | CGAGGAAAATGGTGGGATAA | ChIP |
| LEU 1.4- | ATCGCATTATCCTCGGGTTC | ChIP |
| LEU 1.4+ | TCTGATGGAAGAATGGGCTAA | ChIP |
| LEU 2.4- | CATGCAAGCCACTACACCAA | ChIP |
| LEU 2.4+ | TGTTTCCCAACCGTAAGAGTG | ChIP |
| LEU 6.2- | CCACTACATCATACACACCGACT | ChIP |
| LEU 6.2+ | ACTCCATCGGGAAAAATCCT | ChIP |
| KCC4- | CTCTGGAAATTTCGGTGTCATTG | resection |
| KCC4+ | TCGTATCAGGTCTGCCCTATGAA | resection |
| R0.15- | GAGCAAGACGATGGGGAGTTTC | resection |
| R0.15+ | CCTGGTTTTGGTTTTGTAGAGTGG | resection |
| R0.65- | TCTTATTCGCCTTCTTGGGT | resection |
| R0.65+ | GGAAACACCAAGGGAGAG | resection |
| R0.9- | CGGCATATTTGTATTAACCC | resection |
| R0.9+ | CGATATTAAGTCCTCCGT | resection |
| R1.7- | TTGCTTCACCAATTTTGGAC | resection |
| R1.7+ | TTTGTTTTGCCTAGAAGACTC | resection |
| R3.5- | GGAGCCACAGGATTAATTATC | resection |
| R3.5+ | GAAAGGCGGAATATTGAGGA | resection |
| R6.5- | AGTGAACGCTTGTGCTATCT | resection |
| R6.5+ | GATCATAACGGAGAAGCTAG | resection |
| R8.9- | GAGTATACCTCTATATGCTGTG | resection |
| R8.9+ | AGTGATGACGTACGTGTATAG | resection |
| HO CUT - | TCACCACGTACTTCAGCATA | cutting efficiency |
| HO CUT+ | GTGGCATTACTCCACTTCAA | cutting efficiency |
