# Supplementary figures and images for: The chromatin remodeler Chd1 supports MRX and Exo1 functions in resection of DNA double-strand breaks
Source: PLoS Genet. 2021 Sep 14;17(9):e1009807. doi: 10.1371/journal.pgen.1009807 (PMC8462745; doi:10.1371/journal.pgen.1009807)

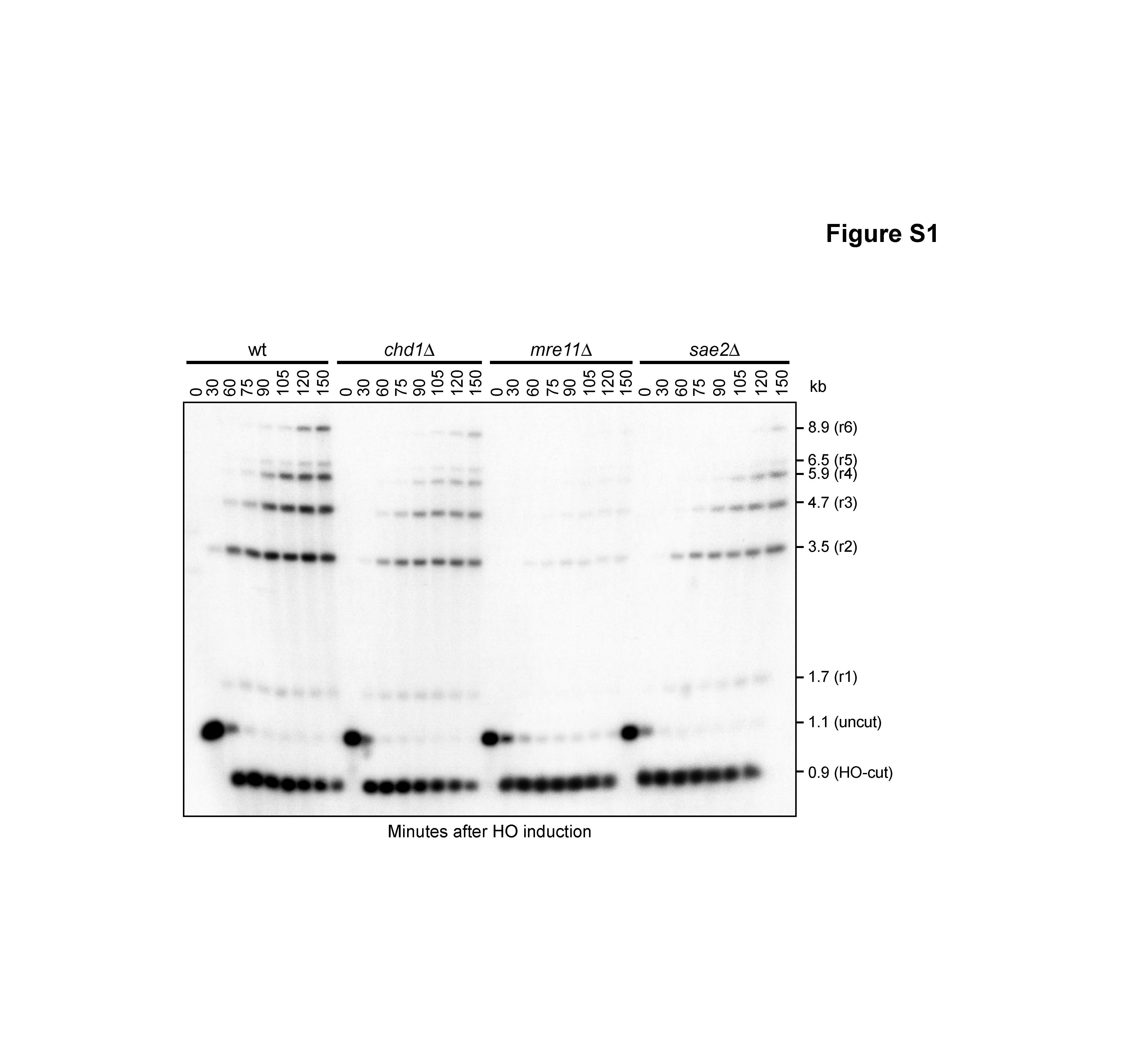

Supplement: S1 Fig — YEPR exponentially growing cell cultures of JKM139 derivative strains were transferred to YEPRG at time zero. Southern blot analysis of SspI-digested genomic DNA after alkaline gel electrophoresis with a probe that anneals to the unresected strand. 5’-3’ resection progressively eliminates SspI sites (S), producing SspI fragments (r1 through r6) detected by the probe. (TIF) [file pgen.1009807.s003.tif]

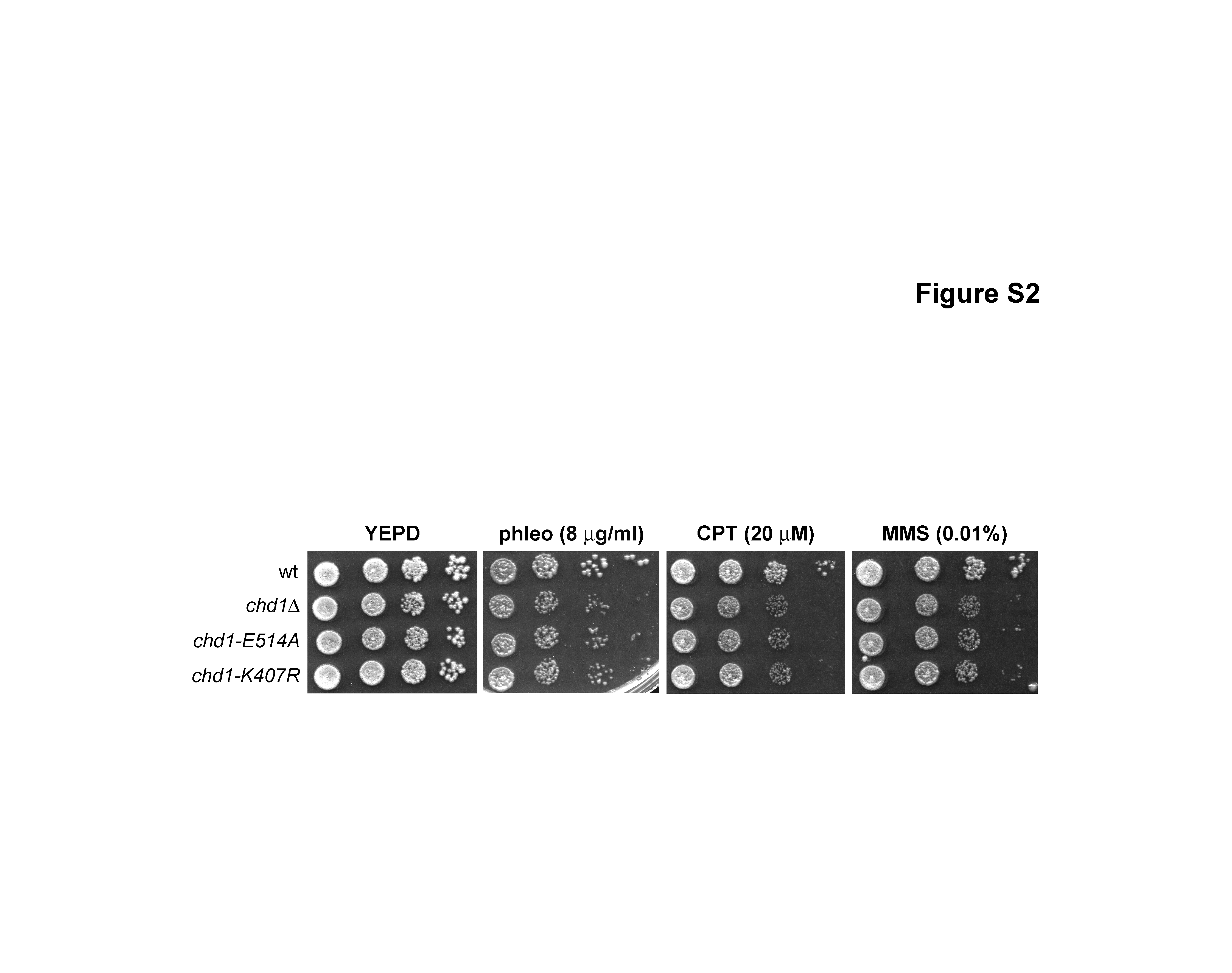

Supplement: S2 Fig — Exponentially growing cultures were serially diluted (1:10) and each dilution was spotted out onto YEPD plates with or without CPT, MMS or phleomycin. (TIF) [file pgen.1009807.s004.tif]
