# Supplementary material for: The chromatin remodeler Chd1 supports MRX and Exo1 functions in resection of DNA double-strand breaks
Source: PLoS Genet. 2021 Sep 14;17(9):e1009807. doi: 10.1371/journal.pgen.1009807 (PMC8462745; doi:10.1371/journal.pgen.1009807)
Supplement: Attachment — Submitted filename: Response to reviewers.docx [file pgen.1009807.s007.docx]

**Response to reviewers’ comments**

**Reviewer #1:**

The paper by Gnugnoli et al suggests that the ATPase activity of the chromatin remodeler Chd1 influences the efficiency of repair by HR, as well as repair by single strand annealing. Both of these require resection, and indeed the loss of Chd1 ATPase activity appears to attenuate long range resection. Moreover, it appears that the efficiency of MRX association with a processed DSB also is influenced by Chd1 at the *MAT* locus. It is unclear whether an initial endonuclease cleavage or only the longer range (>2 kb) resection is influenced by loss of Chd1. Consistent with a direct, rather than indirect effect, the authors show by ChIP that Chd1 is recruited to a DSB, as are a number of other remodelers (notably, RSC, INO80, SWR1 and FUN30 in yeast). We note that the same phenotypes that authors attribute to loss of Chd1 have been attributed to mutants in a number of these remodelers, most significantly INO80 and RSC (reviewed in Chambers and Downs Prog Mol Biol Transl Sci. 2012). It is a shame that the authors do not place their study in context, citing appropriately the large literature that exists on chromatin remodelers in DSB repair. Indeed, the lack of reference to other studies in budding yeast - much of it done 8-10 years ago - is striking, and it weakens the impact of the authors’ novel observations on Chd1. Fortunately, this can be readily remedied by a revision that places the authors’ findings in context (the missing references and concepts will be included below).

The strength of the paper stems from the fact that very few laboratories have studied yeast Chd1 in DSB repair, while in mammalian cells this remodeler (or its family) has been shown to affect HR in a heterochromatic context, as well as stimulating UV-induced damage repair ([reviewed by Rother](https://pubmed.ncbi.nlm.nih.gov/?sort=date&show_snippets=off&term=Rother+MB&cauthor_id=28847822) and [van Attikum](https://pubmed.ncbi.nlm.nih.gov/?sort=date&show_snippets=off&term=van+Attikum+H&cauthor_id=28847822), 2017). Budding yeast has only one Chd like remodeler, while in mammals there are at least 6 (probably reflecting the abundance of heterochromatin in the two organisms). While it is mentioned several times that the human mutation is correlated with prostate cancer, the demonstrated roles in repair are only obliquely mentioned (see papers by Rüthemann et al. 2017, Moore et al. 2019, and Klement et al. 2014, listed below).

Besides failing to position their findings in the field of chromatin remodelers at DSBs, I believe that the presentation of data urgently requires revision prior to publication. First, the normalization of data is not performed appropriately, leading one to believe that the effect of the mutant is stronger than it actually is, and several quantitations (e.g. short range resection) are missing. These are listed below as major points that need to be addressed. In addition, it would be helpful if assays for strand invasion rates were included, to determine if strand invasion is actually delayed in *chd1* ATPase mutants, rather than monitoring the terminal end product of HR mediated repair (NCO vs CO). Drop assays and survival curves following DSB induction are missing, and the use of CPT and MMS to challenge *chd1* mutants seems pointless, given that these trigger completely different pathways of repair. CPT traps Top1 in a covalent ssDNA-protein complex that only converts to a DSB under rare conditions (see Nielsen et al. Nat Methods. 2009) and MMS lesions are repaired by post-replicative repair or TLS, which also do not reflect the pathway of HR-mediated DSB repair. Thus the data on CPT and MMS sensitivity seem out of place. At the very least, Figure 1A and Figure 5 need to be discussed in another context, and survival data following the induction of a DSB should be added.

Despite these weaknesses, the study of the Chd1 remodeler at breaks is interesting and – even if the impact of its loss is less dramatic than currently suggested - a properly revised paper would be a valuable addition to the field.

**Major points to address:**

1. As mentioned above, Figure 1A (drop assays on CPT, MMS and HU) and Figure 5 (CPT, MMS) add almost nothing to a study of DSB resection efficiency (which is the main point of the paper). They could be included as supplemental data: it is unclear why *rad50* *delta* is so different to *rad50-VM,* when combined with *tel1* or *chd1,*  nor why the triple shows epistasis while the double does not (cf. Figure 5A and C). Once again, it is important to note that CPT creates ss-nicks with a protein adduct (not repaired by HR), MMS creates alkylation that is repaired almost exclusively by post-replicative repair mechanisms or TLS (no HR involved) and HU is a fork stalling agent that also does not cause DSBs under normal conditions (fork restart requires Rad51 if breakage occurs but the pathway is distinct from DSB induced HR). It would be helpful if these drop assays were shifted to supplemental data and instead of these, survival curves were included for colony outgrowth after HO-cut induction (for 1-2-4-6h on gal) and replating on glucose containing plates. This can be done with and without a donor sequence (usually placed on another chromosome), and with or without Ku, to eliminate NHEJ. These would be the appropriate assays for monitoring a role for Chd1 in DSB repair.

Fig 1A is now included as Supplementary Fig S2. Furthermore, we deleted Figure 5B-D because of difficult interpretation. We now show percentage of survival for the SSA assay (Fig 4D; Fig. 6F) and the ectopic recombination assay (Fig 5D; Fig 6G).

**2. The most major point**, which affects the key conclusions on resection and recombination efficiency drawn from data in Figure 1C-D, 2B-C, 3B, 4A and 6A,B, is the fact that the authors do not **normalize their data to cut efficiency**. They monitor cut efficiency by monitoring the drop in intensity of the uncut band on the Southern blot, but they never normalize the appearance of the upper bands (resulting from resection) to cut efficiency. The fact that *chd1* mutations reduce cut efficiency is clearly shown in Figure 2B (it is also visible at 60 min in Figure 1C, but the exposure is such that it is harder to see). If cleavage efficiency is reduced by *chd1* mutation, then it is obvious that the appearance of ssDNA will be reduced (Fig 1; 6), and as a result SSA rates will be down (Fig 2). Most likely, also HR products will be down since there are fewer cleavage events to monitor (Fig 3) and certainly histone eviction is strictly dependent on there being a break. Thus with less efficient cleavage, fewer histones will be lost at the *MAT/ LEU2* loci (Figure 4A), exactly what is shown. The question is: how much of the drop is due to CHD1*’s* role in resection, HR or histone eviction, and how much is due to Chd1 being required for HO induction and efficient cleavage ? I would claim that their data – as currently plotted, are inaccurate, because they fail to normalize to the rate of cleavage. This can be remedied, but they do have to quantify the uncut band and in each case, normalize the appearance of the upper band monitored (whether from resection or repair) to the efficiency of cleavage. As said, the fact that *chd1*  mutation reduces cleavage efficiency is clearest in Figure 2 – but every gel actually shows that cleavage efficiency is reduced at early time points (and even at later timepoints). This will not reverse their conclusions but it may make the effects more modest (particularly the attenuated histone removal – see below). For accuracy, however, it is necessary.

All the ChIP data (Mre11, H3, Rad51) had been already normalized to the cut efficiency and this was reported in Materials and methods. We have now normalized to cut efficiency the signals of the resection products (now Fig 2C) and of SSA products (now Fig 4C). The Southern blot to detect CO and NCO events (now Fig 5C) had been already normalized to the cut efficiency, because the intensity of the uncut band at 2 h after HO induction (maximum efficiency of DSB formation) was subtracted to the normalized values of NCO and CO bands at the subsequent time points after galactose addition (see materials and methods). Detection of ssDNA by qPCR have been normalized to the cut efficiency (Figs 2D and 6D).

**3. Figure 1 and Figure 6**, The second most important point, which MAY change the authors’ interpretation of *chd1* effects, is the fact that they do not monitor short-range resection (1.7 kb and less). Resection less than 1.7kb is almost certainly the length that is important for HR-mediated repair (<400-500 nt ssDNA is needed for efficient strand invasion). This has been demonstrated using mutants that interfere with long range (>3-4 kb), but not short-range, resection. In Figure 1D the authors plot the efficiency of resection at 3.5 kb, 4.7 kb, 5.9 kb, 6.5 kb and 8.9 kb, but none of these distances are likely to be relevant for HR. The long-range resection that they observe is important for SSA when the donor is 5-8 kb away, but not for recombination with an ectopic or sister-borne donor. In the Southern blot shown in Figure 1 and 6, they show r1 (1.7kb) but they do not quantify it. It appears that the result for r1 is almost the opposite of that for r2-r6: it *appears* to be relatively elevated in *chd1* delta, rather than lower !

This might be even more pronounced when normalized to cut efficiency. Thus the authors should both normalize by cut efficiency and present the results for short-range resection (0.6kb, 1kb and 1.7kb). Perhaps the authors will find it more easily done with the Diffley lab qPCR assay (Zierhut and Diffley, EMBO J 2008, 27, 1875-85), which is commonly done (for example, Marcomini et al., Cell rep 2018). The reason this may be critical, is because Chd1 appears to contribute primarily to long-range resection. That may explain why it affects SSA repair, more than DSB repair. I note that the Rad51 loading data (Fig 1F) is shown for 0.6kb and 1.8kb, but the resection quantitation only starts at 3.5kb. The drop at 3.5kb is from 22% to 15% in *chd1* delta, but then again this has to be normalized to cut efficiency. Figure 2B shows clearly that the uncut band is nearly twice that of wt in the *chd1* mutants, thus cut efficiency will alter those values.

We have not quantified the r1 resection product because the signal is broad and very low. We have now used the quantitative PCR-based method set up in Diffley’s lab to evaluate generation of restriction enzyme-resistant ssDNA very closed to the HO-induced DSB (Figs 2D and 6D). We found that *CHD1* deletion caused a reduction in ssDNA generation close to the HO cut site (0.15, 0.65, 0.9 kb) (Fig 2D), indicating a defect in the initiation of resection. The same analysis at more distant sites (1.7 and 3.5 kb) (Fig 2D) confirmed the reduction in long-range resection detected by denaturing Southern blotting.

**4. HR assay – Figure 3:** It is advisable that the authors use a qPCR assay to monitor strand invasion quantitatively in the ectopic HR assay shown in Figure 3, to complement the Southern blot. The Southern blot shows the NCO and CO end products, which are lower in the mutants, but it is important to monitor strand invasion, because remodelers may also be aiding the extension of the 3’ end and resolution of the cross over. Thus the data shown are only telling half the story - would be highly important to figure out if – like INO80 – CHD1 is involved in strand invasion and not simply the resolution of HR.

We agree with the reviewer that it is interesting to test whether strand invasion is delayed in *chd1.* However, given that Chd1 dysfunction decreases the efficiency of DSB resection and the association of Rad51 to the DSB ends, the expected delay in strand invasion does not necessarily reflect a role of Chd1 in this process but it could be an indirect consequence of the reduced DSB resection.

**5. Figure 4, Histone depletion near the DSB:**  In Figure 4 the authors show less histone eviction at various distances from a DSB at *LEU2* and *MAT*, yet their data once again are not normalized to cut efficiency. Here it is particularly important because every chromatid that is not cleaved, will retain nucleosomes. Thus if WT cleavage leaves 5% uncut and *chd1*  leaves 15% uncut, yet there is less than 2-fold difference (as shown) in histone occupancy, it may simply be the residual uncut chromatin that retains nucleosomes (and not the effect of CHD1 on eviction). Once again, normalization to cut efficiency is crucial – especially in this experiment (even moreso than in the resection assay).

All the ChIP experiments had been already normalized to cut efficiency, as stated in Materials and methods.

**6. Figure 4C-**F: It is our lab’s experience that anti-Myc and anti-HA (especially anti-HA) precipitate nonspecifically ssDNA– and that during a timecourse of HO induction, the beads with antibodies alone precipitate increasing amount of DNA – as resection increases. For this reason one must always normalize to the no tag pull down at each time pont, and not only to input signal. Thus for all the CHIP experiments using epitope tags, the values should to be normalized to the no-tag control *at each time point*. One can see by the black bars in panels C, E and F that untagged background increases with time (less than the specific pulldown, but still it increases 2-3 fold). Although this may not change the overall results (which are believable for the proteins recovered with high efficiency) it is recommended that the data be normalized to the value of the no tag sample *at each time point*. Since they have the data, it is just a matter of replotting the results. In this case, it will not change the story, but will give more accurate results.

We have modified the graphs of Figs 2F, 3A, 3B, 3E, 5F and 6B, by subtracting the values of the no tag.

**7. Figure 6:** Figure 6 shows the most exciting data of the paper: loss of Chd1 and impaired MRX activity are strongly additive for impaired resection at long distances, but if you look at the 1.7 kb band (r1) it is higher in the double mutant ! And in this instance, cleavage efficiencies in the double *chd1 rad50* and single *chd1* mutants appear to be about the same (although a qPCR quantitation across the break would be helpful- see e.g. Cheblal et al. Mol Cell 2020). This synergy/additivity is strongly divergent from the hypothesis that the *chd1* repair phenotypes derive exclusively from a failure to recruit MRX. It also suggests that there is a difference for short resection (1.7kb) and long resection events (3.5 kb and above). The additivity of the two mutants for resection is opposite of the epistasis seen for *rad50* delta and *chd1* on CPT and MMS (note as I explained above, CPT and MMS repair pathways are not HR). Thus I would definitely stress the interesting single vs double synergy for resection, and check this double for other assays (including cleavage efficiency, ssDNA production by the Diffley assay, and strand invasion by qPCR), and survival of DSB induction (colony outgrowth assay).

We thank the reviewer for this suggestion. We have analyzed in more details MRX association to the HO-induced DSB, DSB resection by qPCR and survival to DSB induction in *rad50-VM cdh1∆* double mutant cells. We found that the amount of Mre11 bound at the HO-induced DSB was lower in *chd1∆* *rad50-VM* cells than in *chd1∆* and *rad50-VM* cells (Fig 6B). Furthermore, ssDNA generation close to the HO cut site (0.15 or 0.65 kb) in *chd1∆* *rad50-VM* cells was similar to that of *chd1∆* cells (Fig 6D). By contrast, a significant reduction in ssDNA generation can be detected in *chd1∆* *rad50-VM* cells compared to *chd1∆* cells at more distant sites (1.7, 3.5, 6.5 or 8.9 kb) (Fig 6D), indicating a more severe long-range resection defect in the double mutant. Consistent with the finding that *chd1∆ rad50-VM* cells compromised extended DSB resection more severely than *chd1∆* cells, viability upon generation of a HO-induced DSB that is repaired by SSA was lower in *chd1∆* *rad50-VM* cells than in *chd1∆* cells (Fig 6F). By contrast, survival upon generation of a HO-induced DSB that is repaired by ectopic recombination was similar in *chd1∆* and *chd1∆ rad50-VM* cells (Fig 6G). We also show that overexpression of *EXO1*, which is the main nuclease involved in long-range resection, partially suppresses the DNA damage sensitivity of both chd1Δ and chd1Δ rad50-VM cells (Fig 6E), supporting a role of Chd1 in promoting Exo1 resection activity.

**Minor points:**

1. In the abstract it is stated that Chd1 function is “crucial to support DNA damage resistance” in MRX mutants – this is not true – on MMS and CPT *rad50* delta is epistatic with *chd1* (Figure 5D).

We have deleted the sentence.

2. Line 66: it is stated “How the replication machinery navigates nucleosomal DNA is not well understood.” Actually, there are 100’s of papers on this (see list of key references below) and even reviews that are quite comprehensive (see also list of reviews). The authors are encouraged to cite J Downs, H van Attikum, and the FUN30 literature - which specifically cover the role of remodelers in resection events. More recently (Challa et al., Mol Cell 2021) the mechanism of histone eviction through INO80 recognition of ub-Histones has been shown to regulate resection. I do recommend a more thorough reading of the literature in this field (listed below).

We have deleted the sentence and cited papers regarding this topic.

3. Line 141 – it is written that Rad50 was equal in all samples but the blot is for Rad51. A typo

We have corrected the typo.

4. Line 68: References 16 – 19 are given as substantiating the role of remodelers in resection. However, they cite papers that do not even address this point, and miss the very first paper that showed clearly that a nucleosome remodeler regulates resection (VanAttikum, Cell 2004, doi: 10.1016/j.cell.2004.11.033.). Of course there have been many additional papers since. The literature cited is sporadic and basically nothing is said about RSC, Fun30 or INO80 – which are major (established) regulators of resection through nucleosomes: RSC (Downs, et al); INO80 (Shen, Peterson, Papamichos, Poli et al), FUN30 (Ira, VanAttikum et al) . The authors are strongly encouraged to read and include in their paper literature about other yeast remodelers in HR and a number of papers on CHD remodelers in mammals. An accurate account of the existing literature is missing. This is not a minor point and these need to be cited accurately:

We have modified the Introduction and mentioned most of the suggested papers.

On mammalian CHD roles in repair:

[Magdalena B Rother](https://pubmed.ncbi.nlm.nih.gov/?sort=date&show_snippets=off&term=Rother+MB&cauthor_id=28847822), [Haico van Attikum](https://pubmed.ncbi.nlm.nih.gov/?sort=date&show_snippets=off&term=van+Attikum+H&cauthor_id=28847822) Philos Trans R Soc Lond B Biol Sci Actions. 2017 Oct 5;372(1731):20160285. DNA repair goes hiphop: SMACA and CHD Chromatin remodellers join the break dance. DOI: [10.1098/rstb.2016.0285](https://doi.org/10.1098/rstb.2016.0285)

Rüthemann P, Balbo Pogliano C, Codilupi T, Garajovà Z, Naegeli H. Chromatin remodeler CHD1 promotes XPC-to-TFIIH handover of nucleosomal UV lesions in nucleotide excision repair. EMBO J. 2017 Nov 15;36(22):3372-3386. doi: 10.15252/embj.201695742.

Klement K, Luijsterburg MS, Pinder JB, Cena CS, Del Nero V, Wintersinger CM, Dellaire G, van Attikum H, Goodarzi AA. Opposing ISWI- and CHD-class chromatin remodeling activities orchestrate heterochromatic DNA repair. J Cell Biol. 2014 Dec 22;207(6):717-33. doi: 10.1083/jcb.201405077.

Moore S, Berger ND, Luijsterburg MS, Piett CG, Stanley FKT, Schräder CU, Fang S, Chan JA, Schriemer DC, Nagel ZD, van Attikum H, Goodarzi AA. The CHD6 chromatin remodeler is an oxidative DNA damage response factor. Nat Commun. 2019 Jan 16;10(1):241. doi: 10.1038/s41467-018-08111-y.

On other yeast nucleosome remodelers in resection and HR:

van Attikum H, Fritsch O, Hohn B, Gasser SM. Recruitment of the INO80 complex by H2A phosphorylation links ATP-dependent chromatin remodeling with DNA double-strand break repair. Cell. 2004 Dec 17;119(6):777-88. doi: 10.1016/j.cell.2004.11.033. PMID: 15607975.

van Attikum H, Fritsch O, Gasser SM. Distinct roles for SWR1 and INO80 chromatin remodeling complexes at chromosomal double-strand breaks. EMBO J. 2007 Sep 19;26(18):4113-25. doi: 10.1038/sj.emboj.7601835. Epub 2007 Aug 30. PMID: 17762868;

Alatwi HE, Downs JA. Removal of H2A.Z by INO80 promotes homologous recombination. EMBO Rep. 2015 Aug;16(8):986-94. doi: 10.15252/embr.201540330. Epub 2015 Jul 3. PMID: 26142279; PMCID: PMC4552491.

Chambers AL, Brownlee PM, Durley SC, Beacham T, Kent NA, Downs JA. The two different isoforms of the RSC chromatin remodeling complex play distinct roles in DNA damage responses. PLoS One. 2012;7(2):e32016. doi: 10.1371/journal.pone.0032016.

Wiest NE, Houghtaling S, Sanchez JC, Tomkinson AE, Osley MA. The SWI/SNF ATP-dependent nucleosome remodeler promotes resection initiation at a DNA double-strand break in yeast. Nucleic Acids Res. 2017 Jun 2;45(10):5887-5900. doi: 10.1093/nar/gkx221. PMID: 28398510; PMCID: PMC5449591.

Chen X, Cui D, Papusha A, Zhang X, Chu CD, Tang J, Chen K, Pan X, Ira G. The Fun30 nucleosome remodeler promotes resection of DSB ends. Nature. 2012 Sep 27;489(7417):576-80. doi: 10.1038/nature11355. Epub 2012 Sep 9.

Costelloe T, Louge R, Tomimatsu N, Mukherjee B, Martini E, Khadaroo B, Dubois K, Wiegant WW, Thierry A, Burma S, van Attikum H, Llorente B.Costelloe T, et al. The yeast Fun30 and human SMARCAD1 chromatin remodellers promote DNA end resection. Nature. 2012 Sep 27;489(7417):581-4. doi: 10.1038/nature11353. Epub 2012 Sep 9.

Valuable reviews on nucleosome remodelers in DSB repair

Seeber A, Hauer M, Gasser SM. Curr Opin Genet Dev. 2013 Apr;23(2):174-84. doi: 10.1016/j.gde.2012.12.008. Epub 2013 Jan 23.

Chambers AL, Downs JA. The RSC and INO80 chromatin-remodeling complexes in DNA double-strand break repair. Prog Mol Biol Transl Sci. 2012;110:229-61. doi: 10.1016/B978-0-12-387665-2.00009-2. PMID: 22749148.

Poli J, et al The INO80 remodeller in transcription, replication and repair. Philos Trans R Soc Lond B Biol Sci. 2017 Oct 5;372(1731):20160290. doi: 10.1098/rstb.2016.0290.

More recent work on mechanistic effects of remodelers in histone eviction (useful methods):

Challa K, Schmid CD, Kitagawa S, Cheblal A, Iesmantavicius V, Seeber A, Amitai A, Seebacher J, Hauer MH, Shimada K, Gasser SM. Damage-induced chromatome dynamics link Ubiquitin ligase and proteasome recruitment to histone loss and efficient DNA repair. Mol Cell. 2021 Feb 18;81(4):811-829.e6. doi: 10.1016/j.molcel.2020.12.021.

Hauer MH, Seeber A, Singh V, et al. Histone degradation in response to DNA damage enhances chromatin dynamics and recombination rates. Nat Struct Mol Biol. 2017 Feb;24(2):99-107. doi: 10.1038/nsmb.3347.

Cheblal A, Challa K, Seeber A et al. . DNA Damage-Induced Nucleosome Depletion Enhances Homology Search Independently of Local Break Movement. Mol Cell. 2020 Oct 15;80(2):311-326.e4. doi: 10.1016/j.molcel.2020.09.002.

**Reviewer #2:**

The authors document that Chd1 has a role in resection of DSB ends and in promoting efficient repair of DSBs. Chd1 is shown to promote MRX binding to DSB ends. Chd1 likely works in the same pathway with MRX as deletion of CHD1 increases the sensitivity of rad50VM mutant (decreased association with DSB) but not with complete deletion of rad50. Finally, Chd1 is shown to help in nucleosome eviction at DSB ends. Together the manuscript presents interesting set of observations, however the authors need to provide additional evidence to support their conclusions.

Comments

Is the effect Chd1 deletion on resection direct or indirect? As chd1 is a chromatin remodeler it is possible that it regulates protein levels of MRX or other resection enzymes. Is Chd1 recruited to DSBs as would be expected if it played a direct role? What is the evidence for direct involvement of Chd1 in resection? Are Mre11, Rad50, and Xrs2 levels and localization similar in wild type and chd1? Genetics indicates that Chd1 works in the same pathway with MRX so at least one could test few things related to MRX levels and localization.

We showed that Chd1 is recruited at the HO-induced DSB (Fig. 1A). Furthermore, *chd1* mutants decreased Mre11 (Fig 3A) and Exo1 (Fig 3B) accumulation at DSBs without affecting their protein levels (Fig 3C and 3D).

It looks that mostly initial resection is impacted in *chd1* mutants. Authors should comment on this and test whether Chd1 affects also extensive resection.

We have now checked short- and long-range-range resection by qPCR. See response to reviewer 1, point 3.

Page 7: Please specify that chd1 cells are only "mildly" sensitive to "high" doses DNA damage inducing agents. In lower doses there is no sensitivity (Fig 6c).

We have modified the sentence.

Page 8-9 “Chd1 promotes DSB repair”

Viability of wt and chd1 cells (tested by plating on YEPGAL plates) in response to DSB needs to be shown for both SSA and ectopic recombination assays to conclude that Chd1 promotes DSB repair. What is shown is only kinetics of repair in nocodazole blocked cells, not the final repair. chd1 mutants are barely sensitive to DNA damaging agents therefore it is surprising that Chd1 is important for the repair of a single DSB. Are chd1 cells sensitive to zeocin, bleomycin or phleomycin?

We have now analyzed viability of wild type and *chd1* mutants in YEPRG plates in strains carrying the SSA (Fig 4D) or the ectopic recombination system (Fig 5D). Furthermore, we added the sensitivity to phleomycin to Fig. 1A (now Fig S1).

In case resection was analyzed in nocodazole blocked cells, at least one set of experiments should be done in growing cells.

All the DSB resection assays have been performed on exponentially growing cells.

Comparison of the sensitivity of chd1, rad50 and double mutant in Figure 5D need to be shown across broad range of drugs concentration to support epistasis.

We have deleted the panel D of Fig 5 according to the comments of reviewer 1.

Other comments

Page 11

Chd1 promotes MRX but not Ku recruitment. Authors conclude that it is consistent with the role of Chd1 in HR and not NHEJ. This should be rephrased as MRX is needed for NHEJ in budding yeast.

We have modified the sentence.

Previously it was reported that Chd1 deficient cells have normal resection PMID: 22960743. This was tested in the same strain background and no decrease of initial or extensive resection was observed. What is the reasons of these different observations? Were the cells arrested here in nocodazole? Methods section directs the reader to a manuscript that potentially indicates G2/M nocodazole arrest. If so it would be important to perform one experiment in growing cells (to exclude unlikely possibility that the effect of CHD1 deletion is specific to G2/M arrested cells). In general it should be indicated whether resection was analyzed in nocodazole blocked cells.

In the mentioned paper, resection was analyzed only at 28 kb from the DSB. As the lack of Chd1 reduces but it does not abolish resection, differences in resection kinetics are difficult to detect when the analysis was performed very far from the DSB.

**Reviewer #3:**

This is a generally clear and careful study of the role of the chromatin remodeler Chd1 in resection of DSBs in yeast. The overall conclusions seem justified, but there are a few places where the paper would benefit from further clarification.

1. The authors conclude that Chd1 plays some role in promoting the association of MRX with DSB ends. Wouldn’t the same results be obtained if the function of Chd1 was to stabilize its association or prevent its removal? Is there a way to see how long MRX says associated with a DSB?

It very hard to discriminate whether Chd1 promotes MRX association to DSBs or prevents its removal. As MRX is recruited at DSBs, we favor the hypothesis that Chd1 promotes its association to DSBs.

2. I always admire the clear denaturing gels published by this lab! However, when I look at the results, it seems to me that r1 (1.7 kb) in the mutants is not that different from WT, suggesting to me that longer-range resection is more affected? So maybe a ChIP or Rad51 at a greater distance would be useful? More on resection below.

We have checked short range resection by qPCR. See response to reviewer 1, point 3.

3. It would be useful in Figs. 1, 2 and 3 to compare these results to mre11∆ or rad50∆ and sae2∆. Is the effect of chd1∆ much less? My impression from Symington or Haber is that deleting MRX delays but doesn’t much reduce HR and even SSA over short distances. For example, Mimitou and Symington’s 2008 SSA results with sae2∆ (Fig. 1) shows a 65% efficiency but little change in kinetics.

We reported a comparison of DSB resection efficiency in *chd1∆*, *sae2∆* and *mre11∆* cells in Supplementary Fig S1. The DSB resection defect of *chd1*∆ is similar to that of *sae2*∆, whereas *mre11*∆ resection defect is more severe.

4. Why a semi-log plot in Fig. 4?

Histone loss is usually reported as Log2 values of the relative enrichment to maximize the differences (see Van Attikum et al., 2007).

5. In the absence of Mre11 or Rad50, is Sae2 ChIP affected? How is Sae2 recruited if not through MRX?

Sae2 recruitment to DSBs does not require MRX (Lisby et al., 2004), possibly because Sae2 can bind directly to dsDNA

6. Again, is chd1∆ rad50-VM less severe than deleting Rad50?

We have measured Mre11 association at the HO-induced DSB in *chd1∆* *rad50-VM* cells. We found that Mre11 binding at the HO-induced DSB was lower in the double mutant than in each single mutant. However, a certain amount of Mre11 was still bound at the DSB, thus explaining the reason why the sensitivity to DNA damaging agents of *chd1∆* *rad50-VM* cells is lower than that of *rad50∆* cells. In any case, we have deleted the epistasis analysis between *chd1∆* and *rad50∆* because it did not add much to the manuscript and was of difficult interpretation.

7. Finally, Chd1 has been implicated in late recombinational steps in meiosis by Matos (i.e. in crossovers). There, Chd1 showed meiosis-specific MutSgamma-Exo1 interactions. These data aren’t discussed, but they raise the possibility that Exo1 might be involved in Chd1’s association mitotically as well.

We have mentioned this paper both in the introduction and in the discussion regarding the role of Chd1 in promoting Exo1 association at DSBs.
