# Supplementary material for: The chromatin remodeler Chd1 supports MRX and Exo1 functions in resection of DNA double-strand breaks
Source: PLoS Genet. 2021 Sep 14;17(9):e1009807. doi: 10.1371/journal.pgen.1009807 (PMC8462745; doi:10.1371/journal.pgen.1009807)
Supplement: Attachment — Submitted filename: Response to reviewers.docx [file pgen.1009807.s008.docx]

**Response to reviewers**

We have now modified the manuscript according to the reviewers’ suggestions as follows:

-We have introduced all the text changes suggested by reviewer 1.

-We have introduced in Table S2 all the primers used in the study (including those used to calculate cut efficiency), as requested by reviewer 3, point 1.

-We have mentioned the paper Peritore et al. 2021 (lines 67-69) and modified the title of line 121, as suggested by reviewer 3, point 2.

-We have mentioned the paper Wild et al. for the CO events (lines 303, 304), as suggested by reviewer 3, point 4.

-We have modified the text (lines 294, 331) according to the suggestion of reviewer 3, point 5.
